# Multiple congenital granular cell epulis in a female newborn: a case report

**DOI:** 10.1186/1752-1947-8-413

**Published:** 2014-12-08

**Authors:** Yun Liang, Yu-Sheng Yang, Yong Zhang

**Affiliations:** Department of Oral and Cranio-maxillofacial Science, Ninth People’s Hospital, Shanghai Jiao Tong University, School of Stomatology, Shanghai Key Laboratory of Stomatology, Shanghai, 200011 China

**Keywords:** Congenital granular cell epulis, Lesions, Newborn

## Abstract

**Introduction:**

Congenital granular cell epulis is an uncommon tumor which is apparent at birth.

**Case presentation:**

Here we report an unusual case of congenital granular cell epulis present in the mouth of a 4-day-old Asian Chinese female newborn. She had six round, soft, multiple, pedunculated swelling masses, of which two were on her upper anterior ridge and four on her lower anterior ridge. The size of the largest lesion was 3.5×3cm, which was causing difficulty in feeding.

**Conclusions:**

The case of a patient with congenital granular cell epulis was reported here because of its rarity. The lesions were surgically removed and satisfactory results were achieved.

## Introduction

Congenital granular cell epulis (CGCE) is an uncommon tumor seen only in the newborn. The gingival overgrowth was first described by Neumann in 1871 [1]. Since then, fewer than 200 cases of CGCE have been reported in the literature to date [2]. New cases were reported in 2014 [3, 4]. Although its histogenesis remains almost unknown, it has been proposed that CGCE originates from epithelial, undifferentiated mesenchymal cells, pericytes, fibroblasts, smooth muscle cells, and nerve-related cells [2]. But for the past several years, there has been sufficient evidence to suggest that the histogenesis of CGCE is most probably neuroectodermal. After reviewing the reported cases of CGCE, Fuhr and Krogh noted that the incidence of this tumor in females was eight times that in males and three times more often on the maxilla than on the mandible [5]. To date, only 15 cases of CGCE have been reported in mainland China and Hong Kong [6]. Here we report a new case of multiple CGCE occurring in a 4-day-old Chinese female newborn who presented with postnatal diagnosis of CGCE and we review the relevant literature. The aim of this case report is to discuss the clinical features, microscopic features, the differential diagnosis and complications of CGCE.

## Case presentation

**Figure 1:**
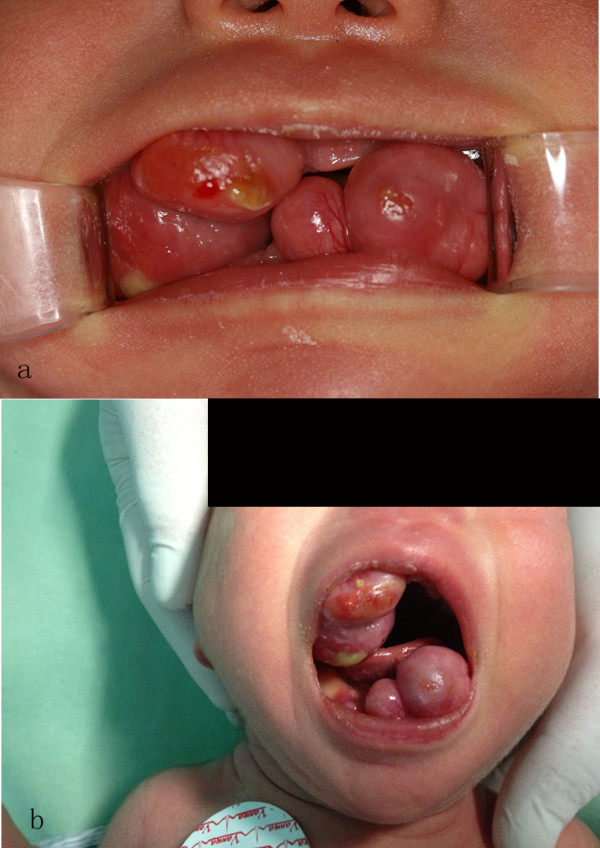
**(a,b) Multiple masses present in the anterior maxillary and mandibular alveolar ridge in a 4-day-old female newborn with congenital granular cell epulis.**

A 4-day-old Asian Chinese female newborn was admitted to our department because she presented with six round, soft, multiple, pedunculated swelling masses with two on her upper anterior ridge and four on her lower anterior ridge. The size of the largest mass was 3.5×3cm (Figure 1). No family history of hereditary diseases was reported. The baby was delivered in the 38th week of gestation via Cesarean. Her birth weight was 2650g. She was unable to close her mouth and thus feeding was not possible. Her respiration was normal. The size of the swelling masses increased slowly after birth. Because of the feeding problems, an immediate surgery was planned. General anesthesia was placed in spare to manage intraoperative complications including blood asphyxiation and other airway-related problems. The feeder vessels were seen to originate from the alveolar ridge. Hence, a transfixion suture was placed slightly away from the lesion on the alveolar ridge so as to achieve pre-excision hemostasis and minimize the chances of intraoperative bleeding, which could endanger the airway.

All lesions were well defined, firm, round, smooth and pink in color on the cut surface. All alveolar masses were excised surgically without complications under general anesthesia on the sixth day after the birth of the baby, who was discharged on the third postoperative day. Postoperative recovery and surgical site healing were satisfactory.

The excised masses were fixed in 10% neutral buffered formalin. The tissue was submitted for histopathological examination. Immunohistochemical analyses were also carried out using a panel of antibodies, including vimentin, Ki-67, smooth muscle actin (SMA), synuclein (Syn), neuron-specific enolase (NSE) and S-100. Corresponding positive and negative controls were performed in parallel for all the antibodies tested.

**Figure 2:**
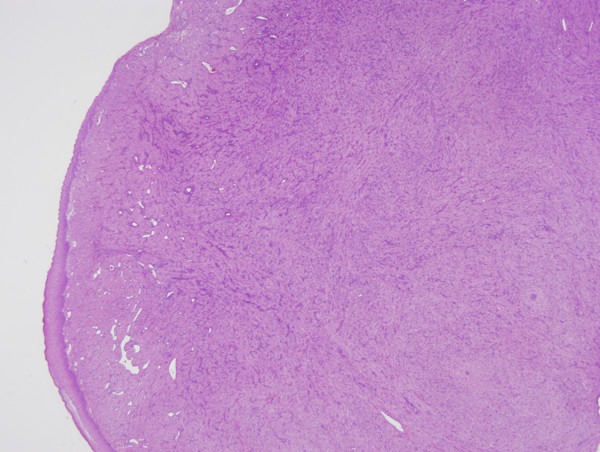
**Lesional cells are compactly arranged with indistinct cytoplasmic outline (hematoxylin and eosin stained, magnification 20×).**

**Figure 3:**
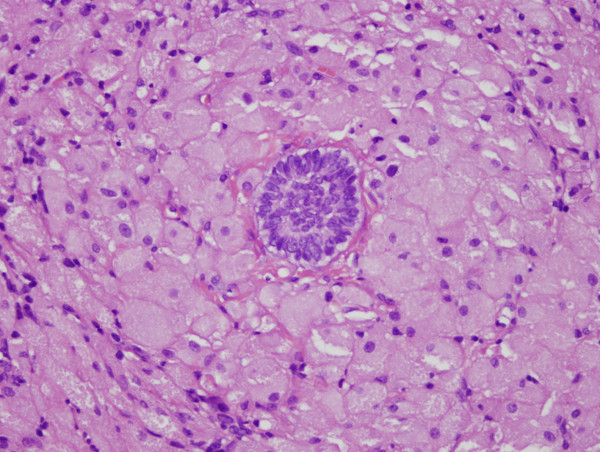
**Closely packed polygonal cells with granular cytoplasm and small round regular nuclei with inconspicuous nucleoli (hematoxylin and eosin stained, magnification 400×).**

Microscopic examination showed a benign tumor composed of sheets of closely packed, large, rounded polygonal cells with abundant granular, eosinophilic cytoplasm and round to oval and lightly basophilic nuclei (Figure 2). The overlying mucosa showed a well-differentiated, stratified squamous epithelium (Figure 3). The tumor was stained diffusely but strongly for vimentin and NSE, and was focally but weakly positive for Ki-67 and negative for SMA, Syn and S-100 protein. The diagnostic hypothesis of congenital epulis of the newborn was confirmed based on both the histological details and immunohistochemical profile of the masses.

**Figure 4:**
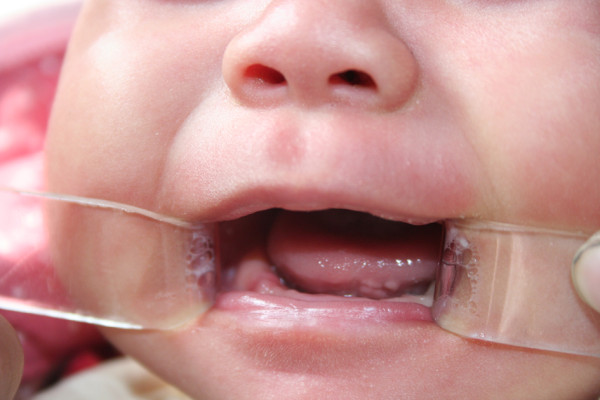
**2 months follow-up.**

Follow-up was conducted for the next 2 months of the baby’s life; no signs of recurrence were found (Figure 4).

## Conclusions

CGCE is an uncommon lesion arising from the mucosa of the gingiva, typically from the anterior part of the maxillary alveolar ridge, but some cases have also been reported in the mandibular gingiva where the lesion was localized (with the 3:1 ratio of maxillary to mandibular predilection) [7–10]. It has an 8:1 ratio of female: male preponderance. Of the 15 cases of CGEC occurring in mainland China and Hong Kong, 13 were females [6]. The female gender of the patient presented in this case report is consistent with the sex predilection of CGCE. While an endogenous hormonal influence has been proposed to explain this gender bias, this is not supported by this case because no estrogen and progesterone receptors were detected within the lesion [2]. Another explanation for this female preponderance is the possibility of an intrauterine stimulus from the fetal ovaries [11]. This is in consonance with most cases that are pedunculated. However, a few sessile cases have been reported. About 90% of CGCE cases are solitary, but not in this neonate [8]. Antenatal ultrasonographic diagnoses of CGCE have been described in the literature. The earliest reported case was identified in a 31-week fetus by ultrasound [12].

The lesions were excised under general anesthesia because they were initially thought to be malignant neoplasm. Reported cases have been excised under either local anesthesia or general anesthesia [8, 13]. CGCE is not prone to recurrence even if some tissue residues are left. No cases of recurrence have been documented in the literature to date.

The diagnosis of CGCE should essentially be clinical, but it could pose some difficulties as seen in this case because of the low level of suspicion. While CGCE is uncommon, it is important that dentists should be able to recognize it as they may be asked to consult and provide crucial information on patient management as well as allay the anxiety of parents. Polyhydramnios is a common symptom in patients with CGCE due to swallowing difficulties as a result of the large size of the mass growth, but the mother did not attend antenatal clinics and gave birth at home.

On histological examination, CGCE is very similar to granular cell tumor (GCT) but different from GCT epidemiologically as well as in clinical behaviors. CGCE is seen exclusively on neonatal gingivae, presents at birth and has a marked predilection for females, whereas GCT is rarely seen in the first decade of life, it is most frequently diagnosed between the third and the sixth decades of life, affects a wide variety of visceral and cutaneous sites, and also has a predilection for the female gender [12].

However, a particularly interesting finding made in this study is the demonstration of a strongly positive immune staining with NSE and a negative staining for S-100 protein. In contrast to GCT, the overlying epithelium never shows pseudoepitheliomatous hyperplasia but typically demonstrates atrophy of the rete ridges. In addition, in contrast to GCT, immunohistochemical analysis showed that the tumor cells were negative for S-100 protein, whereas GCT usually demonstrates a strong staining for S-100.

The histogenesis of CGCE is not clear, and proposed derivations include odontogenic epithelium, undifferentiated mesenchymal cells, pericytes, fibroblasts, smooth muscle cells, nerve-related cells, and histiocytes [8, 9]. However, it is currently accepted that the epulis represents a reactive entity, and recent immunohistochemical staining and ultrastructural examination favor myofibroblasts as the cells of origin.

Recommended management for epulis is surgical excision, although some opt to wait for spontaneous regression if the mass is small and does not interfere with respiration or feeding. In addition to simple excision, the techniques of undermining and advancing gingivoperiosteal flaps, and suturing them over the bony defect, and thus extrapolating for cleft alveolar management have also been reported by Millard and Latham [14].

## Consent

Written informed consent was obtained from the patient’s legal guardian for publication of this case report and any accompanying images. A copy of the written consent is available for review by the Editor-in-Chief of this journal.

## Abbreviations

CGCE: Congenital granular cell epulis
GCT: Granular cell tumor
NSE: Neuron-specific enolase
SMA: Smooth muscle actin
Syn: Synuclein.
